# A Novel and Convenient Method for the Preparation and Activation of PRP without Any Additives: Temperature Controlled PRP

**DOI:** 10.1155/2018/1761865

**Published:** 2018-05-13

**Authors:** Lijuan Du, Yong Miao, Xin Li, Panli Shi, Zhiqi Hu

**Affiliations:** Department of Plastic and Aesthetic Surgery, Nanfang Hospital of Southern Medical University, Guangzhou, China

## Abstract

Platelet rich plasma (PRP) is a concentrate of autologous platelets which contain enrichment growth factors (GFs). However, the addition of exogenous anticoagulant and procoagulant may result in clinical side effects and raise the price of PRP. Herein, we report a novel method named temperature controlled PRP (t-PRP), in which exogenous additives are dispensable in the preparation and activation process. Human blood samples were processed by a two-step centrifugation process under hypothermic conditions (4°C) to obtain t-PRP and rewarming up to 37°C to activate t-PRP. Contemporary PRP (c-PRP) was processed as the control. t-PRP showed a physiological pH value between 7.46 and 7.48 and up to 6.58 ± 0.45-fold significantly higher platelet concentration than that of whole blood compared with c-PRP (4.06-fold) in the preparation process. Meanwhile, t-PRP also maintained a stable GF level between plasma and PRP. After activation, t-PRP demonstrated natural fiber scaffolding, which trapped more platelet and GFs, and exhibited a slow release and degradation rate of GFs. In addition, t-PRP exhibited the function of promoting wound healing. t-PRP is a novel and convenient method for the preparation and activation of PRP without any additives. Compared to c-PRP, t-PRP reflects more physiologic characteristics while maintaining high quality.

## 1. Introduction

Platelet rich plasma (PRP) is concentrate of platelets from autologous blood. Growth factors (GFs) including PDGF (platelet derived growth factor), VEGF (vascular endothelial growth factor), FGF (basic fibroblast growth factor), EGF (epidermal growth factor), IGF (insulin-like growth factor), and TGF-*β*1 (transforming growth factor *β*1) are released from the alpha granules of concentrated platelets activated by coagulants [1–3]. Platelet derived GFs are not only known to enhance recruitment, proliferation, and differentiation of cells, but also thought to play a role in angiogenesis and inflammation. PRP has been used in various surgical procedures and clinical treatments, especially in the field of plastic surgery and dermatology, due to its effects of promoting wound healing, grafted fat survival, and hair growth [4–7].

Thus far, the preparation method of PRP has been reported in several articles with different procedures. Although the yield and vitality of platelets vary in different preparation procedures, the typical preparation and application procedure of PRP are common. First, anticoagulants such as ACD-A (anticoagulant citrate dextrose solution, solution A) is added to the whole blood for anticoagulation [8–13]. Second, coagulants such as calcium gluconate and thrombin are added to activate PRP [14–17]. However, these exogenous additives may have side effects in clinical setting. For example, sodium citrate may cause aggregation of platelets, resulting in inaccurate platelet counts that impact the clinical outcome [12, 18, 19]. Meanwhile, xenogeneic thrombin and other coagulants may trigger immunogenic reactions [20, 21]. On the other hand, due to the presence of anticoagulants, an additional thrombin mixing and spraying kit were further required after using a commercial PRP preparation kit, during the PRP application, especially in wound repair. The use of these commercial kits will undoubtedly increase the clinical cost of PRP. To ensure clinical effectiveness and availability, less exogenous substances are preferable when applying PRP in the clinical setting. Therefore, it would be ideal to prepare PRP without any requirement for additives.

**Figure 1:**
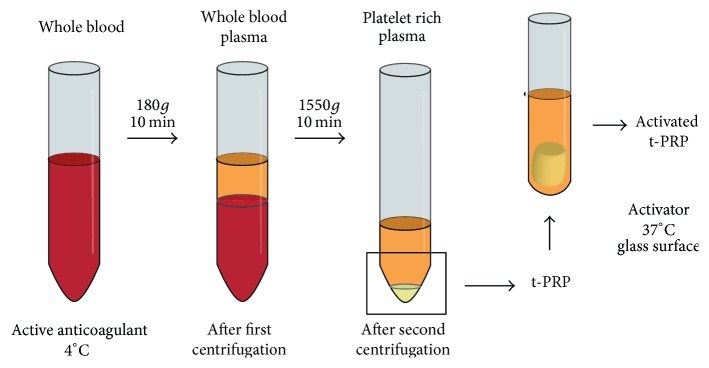
*Schematic representation of temperature controlled PRP preparation*. Venous blood was collected and passed through a two-step centrifugation process by the temperature controlled method to obtain t-PRP.

Herein, we report a novel method of PRP preparation-activation, by which the coagulation was inhibited previously in hypothermic environment and then the activation of platelets through autologous thrombin activity restoration followed only by rewarming. It means this method does not require anticoagulants or coagulants (Figure 1). Temperature controlled PRP (t-PRP) can achieve much better physiologic effects while guaranteeing high quality, compared to contemporary PRP (c-PRP). This modification may provide more accurate platelet counts, with further improvements in safety and clinical use.

## 2. Methods

### 2.1. Samples Collection

Venous blood was obtained from 40 healthy adult volunteers (age 26 to 36 years) after obtaining informed consent. For each individual, half of the blood was processed as t-PRP and the other half was processed as c-PRP.

### 2.2. Preparation of Temperature Controlled PRP (t-PRP)

In order to maintain the stability of t-PRP, the process must be performed under hypothermic conditions using precooled tubes and tips. 10 ml of fresh whole blood was added in 15 ml centrifugation tubes (Corning, Corning, NY, USA) and centrifuged at 200*g* for 10 min at 4°C. Subsequently, the plasma was transferred to new tubes and centrifuged again at 1550*g* for 10 min at 4°C. One milliliter of plasma at the bottom and precipitated platelets were used as t-PRP. The t-PRP was transferred to a new glass tube and then incubated for 15 min at 37°C and then served as activated t-PRP. The control group, c-PRP, underwent a two-step centrifugation process at room temperature containing anticoagulation by ACD-A (Citra Labs, Braintree, MA, USA) and it was activated by thrombin (Sigma, St. Louis, MO, USA) as described previously [22].

### 2.3. Platelet Count and pH Value Analysis of Blood Samples

To analyze the effect of t-PRP preparation, we measured platelet concentration and pH of whole blood, plasma after first centrifugation, and PRP in both t-PRP and c-PRP groups. Blood samples were tested immediately on the Sartorius PB-10 pH analyzer (Sartorius, Goettingen, Germany). Blood samples collected in K_3_-EDTA type anticoagulant tubes (BD Vacutainer®, Franklin Lakes, NJ, USA) were tested on the Sysmex XE-2100 hematology analyzer (Sysmex, Kobe, Japan) after at least 1 h of shaking to avoid platelet aggregation.

### 2.4. Histologic Observation

For hematoxylin and eosin (H&E) and Masson staining, the c-PRP and t-PRP gels were fixed in 4% paraformaldehyde and embedded in paraffin. Paraffin-embedded skin tissues were cut at 5 *μ*m intervals. HE and Masson staining were performed using standard protocols.

For transmission electron microscopy (TEM) analysis, the c-PRP and t-PRP gels were fixed with 2.5% glutaraldehyde. Super-thin sections were prepared and stained with uranyl acetate and lead citrate and were further analyzed using a Tecnai-10 TEM microscopy (Philips, Amsterdam, Netherlands).

For scanning electron microscopy (SEM) analysis, the c-PRP and t-PRP gels were fixed with 2.5% glutaraldehyde and then dehydrated in concentrated gradient alcohol. Then, the samples were coated with gold-spraying under vacuum conditions before characterization by S-3000N SEM microscope (HITACHI Company, Tokyo, Japan).

### 2.5. Protein Quantification Analysis

To determine the amount of contained GFs during each step of preparation process between c-PRP and t-PRP groups, 1 ml of blood sample was concomitantly collected and frozen.

We determined the amount of GFs released from the PRP gel at 0.5, 2, 4, 6, 12, 24, 48, 72, and 96 h time points. The c-PRP and t-PRP gels were incubated with 1 ml of phosphate-buffered saline (PBS) at 37°C. At each time point, 1 ml of conditioned PBS was collected, frozen, and replaced with a new 1 ml of PBS.

We then determined the degradation of released GFs in activated PRP at 0.5, 2, 4, 6, 12, 24, 48, 72, and 96 h. Ten milliliters of activated c-PRP and t-PRP were incubated at 37°C. At every time point, 1 ml of PRP was also collected and frozen.

All samples and standards were assayed in duplicate and mean values were calculated. The results were multiplied by the dilution factor applied to the samples. Enzyme-linked immunosorbent assay (ELISA) kits (R&D Systems, Minneapolis, MN, USA) were used to determine the amount of VEGFs, PDGFs-AB, PDGFs-BB, TGFs-*β*1, bFGFs, EGFs, and IGFs. Absorbance was measured using a multiscan MK3 plate reader (Thermo Fisher Scientific, New York, NY, USA). To determine the amount of contained GFs between the c-PRP and t-PRP gels, western blot analysis was performed. Primary antibodies were incubated as follows: PDGFs-A, PDGFs-B (Bioss, Woburn, MA, USA), and VEGFs (Abcam, Cambridge, MA, USA). Finally, immune complexes were detected by an enhanced chemiluminescence kit (Thermo Fisher Scientific, USA). Results were further quantified using Image-Pro Plus 6.0 software (Media Cybernetics, Silver Springs, MD, USA).

### 2.6. Evaluation of the Function of t-PRP* In Vivo*

Adult male BALb/c nude mice (7 weeks old) were purchased from the Experimental Animal Centre of the Southern Medical University (Guangzhou, China). All animal experiments were carried out under the approval of the Institutional Animal Care and Use Committee from the Experimental Animal Welfare and Ethics Management Committee of the Southern Medical University (Guangzhou, China). All procedures involving animals were performed in accordance with the relevant guidelines and regulations.

To investigate the effect of t-PRP on wound healing, a 1 cm diameter full thickness skin wound was created on the dorsal skin of the nude mice as previously described [23]. Two hundred microliters of t-PRP or PBS was transplanted, respectively, onto the wound. After the t-PRP gelatinized, sterile dressings were applied to ensure incorporation of the graft with the host skin. One week later, the sutures and protective silicone layer were removed. The wound was monitored for 3 weeks.

### 2.7. Statistical Analysis

Two-way analysis of variance was used for statistical analysis, with a *p* value less than 0.05 considered statistically significant. Experimental data were expressed as the means + standard error of the mean. All experiments were repeated at least three times.

## 3. Results

### 3.1. Characteristic Identification of Platelet Enrichment Procedure

**Figure 2:**
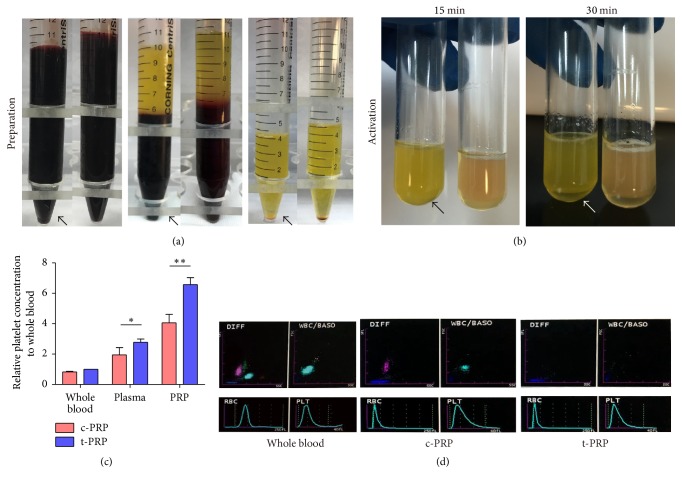
*Comparison of the platelet concentration procedure between t-PRP and c-PRP*. (a) Photographs showing the preparation and activation process of PRP between t-PRP and c-PRP. Whole blood (left), whole blood after the first centrifugation (middle), and plasma after the second centrifugation (right). Compared to c-PRP, the t-PRP group more clearly shows the boundary between red blood cells and plasma and the absence of red blood cell contamination in the plasma and PRP. (b) After activation, t-PRP completely gelled in about 15 min and began to precipitate plasma until 30 min. The arrow indicates the t-PRP group. (c) The relative concentration of platelets to whole blood between t-PRP and c-PRP groups. t-PRP showed significantly higher platelet counts than c-PRP. (d) Hematology analysis showed that, compared with whole blood (left) and c-PRP (middle), t-PRP (right) had a marked decrease in red blood cells and leukocytes while remaining unchanged in platelet distribution width and peak. ^*∗*^*p* < 0.05; ^*∗∗*^*p* < 0.01.

After first centrifugation, t-PRP showed a clear boundary between red blood cells and plasma and a thin buffy coat. In contrast, c-PRP showed an indistinct boundary. The resulting plasma and PRP in the t-PRP group were clearer and had less red blood cell contamination (Figure 2(a)). After activation, the t-PRP gelling process was slow and complete in about 15 min and plasma began to precipitate until 30 min, while c-PRP gelled immediately after adding thrombin (Figure 2(b)).

Platelet number, concentration, and pH of the t-PRP and c-PRP groups are summarized in Table 1. t-PRP showed a physiological pH between 7.46 and 7.48, while c-PRP was more acidic (pH 7.07–7.10). Meanwhile, in the whole blood, plasma, and PRP, the platelet concentration of t-PRP was significantly higher than those of c-PRP (Figure 2(c)). Notably, the platelets concentration of PRP in the t-PRP group was up to 6.58 ± 0.45-fold higher than that of whole blood, while that in the c-PRP group was increased only 4.06 ± 0.55-fold. t-PRP removed a large number of red blood cells and leukocytes, and the platelet distribution width and peak did not change significantly compared with whole blood and c-PRP (Figure 2(d)).

### 3.2. Histological Analysis of PRP Gel

**Figure 3:**
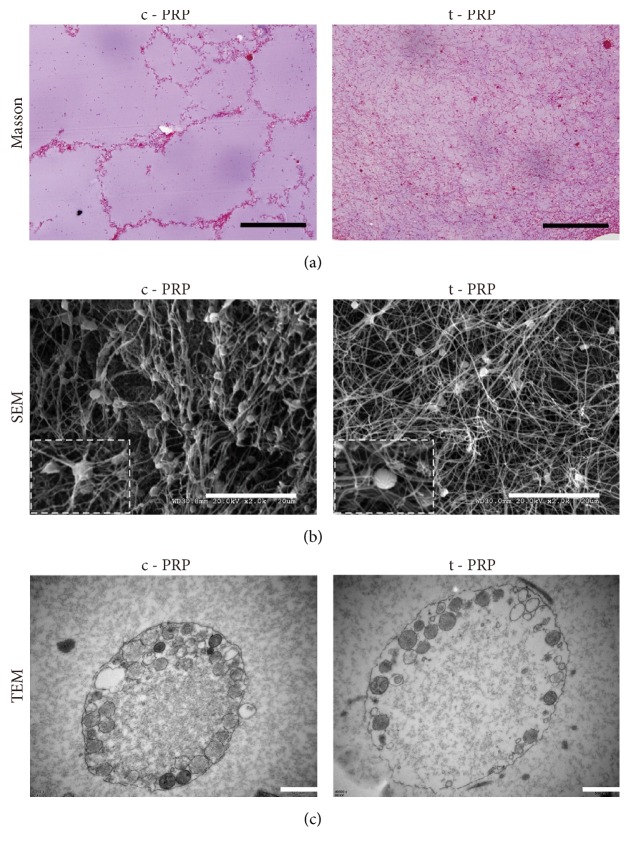
*Histologic observations between t-PRP and c-PRP*. (a) Masson staining of both c-PRP and t-PRP gels. The c-PRP gel showed a more tightly compressed fibrin network and more uneven platelet aggregation than t-PRP. Scale bars = 100 *μ*m. (b) SEM analysis of the PRP gel showed that the fibrin networks in the c-PRP gel were constituted by the thick and tightly packed fibrin polymers, while the t-PRP gel was looser and uniformly distributed. Scale bars = 20 *μ*m. Magnification SEM view showed that the platelets were trapped in the fibrin mesh. (c) TEM analysis showed that platelets from both groups were activated and degranulated. Scale bars = 50 nm.

The c-PRP gel formed by thrombin showed dense or tightly compressed fibrin networks with minimal interfibrous space. Platelets were observed unevenly or were partially aggregated in the gel (Figure 3(a), left). In the t-PRP gel, a uniform and looser structure was seen with more interfibrous space and more platelets could be observed to be cross-linked in the fiber networks. Furthermore, the platelets were more evenly distributed compared to the c-PRP gel (Figure 3(a), right). SEM analysis confirmed that the fibrin networks in the c-PRP gel were constituted by thick and tightly packed fibrin polymers, which led to the constitution of rigid networks (Figure 3(b), left). In contrast, fibrin in t-PRP has polymerized naturally and slowly, which allows the establishment of fine and flexible fibrin networks that are able to enmesh platelets and cytokine (Figure 3(b), right). Meanwhile, the TEM analysis demonstrated that 37°C rewarming could also activate and degranulate platelets of t-PRP, which has the same effect as adding thrombin to c-PRP (Figure 3(c)).

### 3.3. Quantification Analysis of GFs Concentration, Release, and Degradation

**Figure 4:**
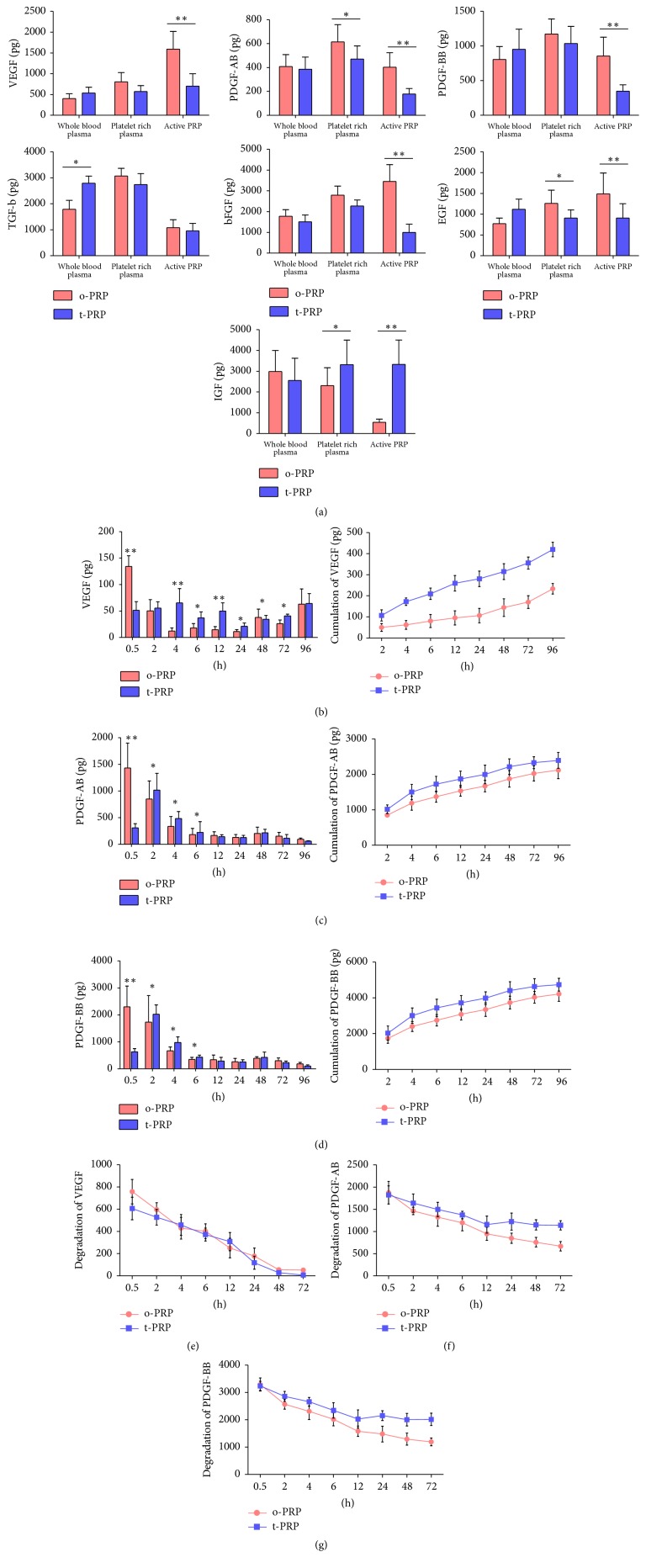
*ELISA protein quantification of GF of t-PRP and c-PRP groups*. (a) GF quantification of VEGF, PDGF-AB, PDGF-BB, bFGF, EGF, and IGF in plasma, PRP, and activated PRP. Compared to c-PRP, t-PRP showed a relative stabile content of GFs in plasma and PRP, which then decreased greatly in the plasma after activation. (b–d) GFs released from PRP gel quantification at each time point of VEGF ((b), left), PDGF-AB ((c), left), and PDGF-BB ((d), left) over a 96 h period. Total accumulation of GFs over 0.5–96 h for VEGF ((b), right), PDGF-AB ((c), right), and PDGF-BB ((d), right). The release rate of GFs decreased greatly with time in the c-PRP group, while t-PRP showed slower release characteristics. (e–g) GFs degraded in activated PRP quantification at each time point of VEGF (e), PDGF-AB (f), and PDGF-BB (g) over a 72 h period. VEGF was not significantly different between groups, whereas PDGF-AB and BB degraded slowly in t-PRP. ^*∗*^*p* < 0.05; ^*∗∗*^*p* < 0.01.

To verify the efficiency of the temperature controlled method, the release of GFs including VEGF, PDGF-AB, PDGF-BB, TGF-*β*, bFGF, EGF, and IGF that are known to be important signaling molecules in tissue repair [24] was examined between t-PRP and C-PRP groups (Figure 4(a)). We found that most of the GFs content was not significantly different between two groups in plasma and PRP before activation. Meanwhile, t-PRP had more stable GFs content during the enrichment process. This finding confirmed the fact that hypothermic condition could maintain platelet stability. Interestingly, in the plasma of activated t-PRP, several GFs were unexpectedly decreased greatly, except for IGF.

These results raised the possibility that platelet derived GFs may be trapped in the fibrin meshes of t-PRP more than c-PRP after activation. To validate this theory, we analyzed three of the most important GFs (PDGF-AB, PDGF-BB, and VEGF), which we performed at each time point as well as the accumulation over time. We found that after 0.5 h of activation significantly higher level of VEGF was released from c-PRP compared to t-PRP. However, the release of VEGF in c-PRP dropped rapidly, while t-PRP still showed an upward trend until 4 h (Figure 4(b), left). The total accumulation of VEGF demonstrated that t-PRP had significantly higher level than c-PRP (Figure 4(b), right). A similar releasing curve was observed in PDGF-AB and PDGF-BB, where c-PRP peaked at 0.5 h and then dropped, and the t-PRP group showed a statistically higher level of these two factors from 2 h to 6 h (Figures 4(c) and 4(d)). Meanwhile, the degradation analysis of GFs released in plasma (free-GFs) was performed. The free-VEGF degradation rate of t-PRP was slower than c-PRP during the first 12 h, and it tends to be consistent over time (12–72 h) (Figure 4(e)). The free-PDGF-AB and PDGF-BB degradation curve showed a significantly slower degradation rate in t-PRP than c-PRP over time. At the end point of 72 h, only 1/3 of free-PDGF-AB and PDGF-BB was left in c-PRP, but in t-PRP about 2/3 remained (Figures 4(f) and 4(g)). Rapid degradation of free-GFs was observed in both groups, but a relatively slower rate was observed in t-PRP group.

**Figure 5:**
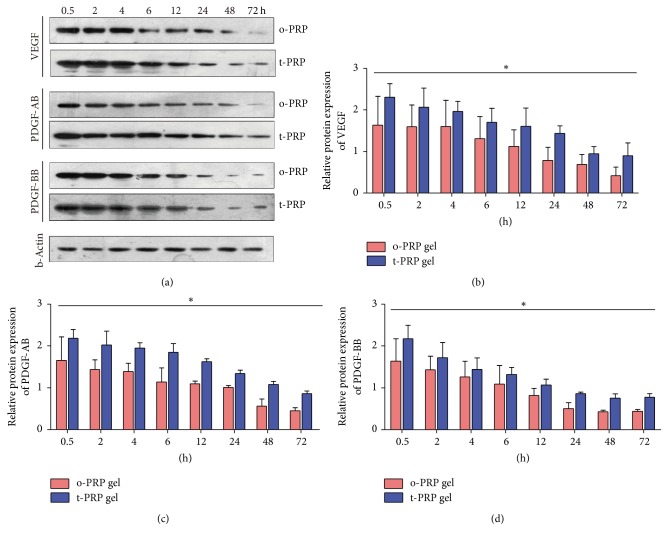
*Western blot protein quantification of GF content in the PRP gels*. (a–d) GF quantification in the PRP gel of VEGF (b), PDGF-AB (c), and PDGF-BB (d) at each time point over a 72 h period. Compared to c-PRP, VEGF, PDGF-AB, and BB showed a significantly higher content in t-PRP at all time points. ^*∗*^*p* < 0.05.

To test strength of the theory that GFs become more trapped in the fibrin meshes of t-PRP, we analyzed GF content in the gel at each time point. The residual GFs in the gel showed a significantly higher expression of VEGF, PDGF-AB, and PDGF-BB in t-PRP compared with c-PRP (Figure 5(a)). The residual VEGF, PDGF-AB, and PDGF-BB in the t-PRP group were observed to exceed 30%, compared with the c-PRP group, during the first 4 h; this further increased, reaching approximately 2-fold at 72 h (Figures 5(b)–5(d)).

When considering release and degradation, t-PRP appears to be able to preserve more GFs over time while gradually releasing GFs, compared with the rapid degradation in the plasma of c-PRP.

### 3.4. t-PRP Promoted Wound Healing in an Animal Model

**Figure 6:**
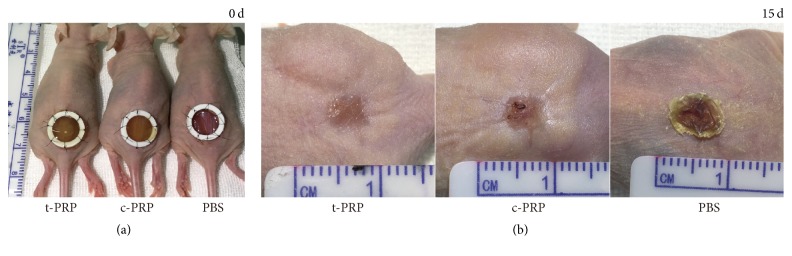
*PRP promoted wound healing in vivo*. (a) Gross examination showed that the wound healing model of nude mice with time in between t-PRP, c-PRP, and PBS control. (b) The t-PRP and c-PRP groups were completely reepithelialized, while the control group demonstrated large crusts in the wound surface by day 15.

To assess the efficacy of t-PRP on wound healing, a full thickness wound was created on the backs of nude mice and treated with t-PRP, c-PRP, and PBS, respectively (Figure 6(a)). After 15 min, t-PRP had gelatinized completely on the wound while only a small amount of c-PRP gel was formed. This finding indicates that, compared with c-PRP, t-PRP did not require additional thrombin and spraying equipment, which can be quickly activated into gel and cover the wound. Fifteen days after surgery, the wounds in both t-PRP and c-PRP treatment group were reepithelialized completely, without obvious crust formation during the process (Figure 6(b)). These findings suggest that t-PRP has a similar function of promoting wound healing to c-PRP* in vivo*, as previously reported [1, 25, 26].

## 4. Discussion

In this study, a new platelet enrichment procedure was performed, which only used a temperature controlled method at 4°C to inhibit blood clotting and 37°C rewarming to activate plasminogen and platelets, which required neither anticoagulants nor bovine thrombin.

Many studies have reported that cold storage of platelets at 4°C acquires better metabolic results, such as minimal lactate accumulation, aggregation response, and adhesion to the subendothelium [27, 28]. However, platelet refrigeration has been abandoned in blood transfusion because it causes a shorter circulation time* in vivo* [29, 30]. In our study, we demonstrated that 4°C conditions play a positive role in replacing ACD-A to inhibit blood clotting and maintain platelet morphology and functional integrity. Considering that the usage of t-PRP is different from blood transfusion, where it is unnecessary for the platelets to enter circulatory system, the disadvantages of 4°C storage can be neglected in this study. In addition, due to the absence of anticoagulants, t-PRP can be activated rapidly after a rewarming process* in vitro* by autologous thrombin or after injection* in vivo*.

Compared to other platelet concentrate studies, the characteristics of t-PRP include those of both c-PRP and platelet rich fibrin matrix (PRFM), with each having its own unique advantages. t-PRP has an enrichment process similar to c-PRP, which obtained its platelet concentration by a two-step centrifugation. In our study, we choose a low centrifugation speed (200*g*) at the first centrifugation which could reduce cell pull-down so that the majority of platelets will be left within the plasma, separating from the leukocytes in the buffy coat [10]. However, under the same centrifugation conditions, c-PRP demonstrated (1) a relatively indistinct boundary and buffy coat, (2) obtaining less quantity of platelets [18], and (3) a higher level of GFs contents in PRP than plasma, which implies that ACD-A anticoagulants may destroy a part of the blood cells (including red blood cells and platelets). This may be due to a decrease of extracellular calcium ions and pH caused by citrate based anticoagulants, which reduces the stability of the cell membrane and leads to the destruction of senescent blood cells by centrifugal force. In contrast, t-PRP under cryopreservation exhibits a more physiologic pH, higher platelets yield, and slower release and degradation of growth factors.

**Figure 7:**
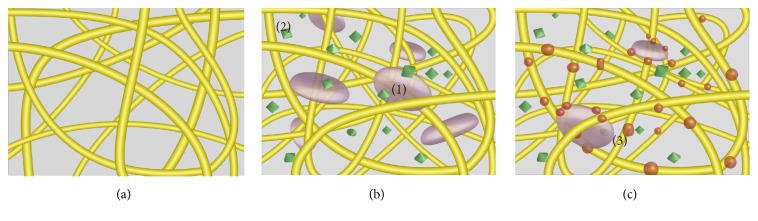
*Theoretical computer modeling of a t-PRP gel*. (a) Theoretical computer modeling of fibrin networks resulting from fibrin glue polymerization. (b-c) Theoretical computer modeling of fibrin networks resulting from c-PRP (b) and t-PRP (c) polymerization. The c-PRP showed that activated platelets are trapped in the fibrin meshes and release GFs extrinsically retained in the fibrin architecture. The t-PRP slow polymerization process would allow the intrinsic retaining of platelet derived GFs within fibrin polymers. (1) Platelet trapped in the fibrin gel. (2) Platelet derived GFs in solution. (c) Fibrin networks resulting from t-PRP polymerization. (3) Platelet derived GFs trapped in fibrin gel.

On the other hand, the gelling principle and structures of t-PRP are similar to PRFM (PRFM is obtained by one-step centrifugation at room temperature without anticoagulants) in that activation process which depends on the physiologic concentration of thrombin in autologous blood [31–36]. The fibrin networks thus formed under a natural and flexible polymerization mode, which increased incorporation of released platelet derived GFs in the fibrin mesh [37–40]. This configuration implies an increased lifespan better maintained and available in situ for these GFs (Figure 7). However, PRFM is a fibrin clot unable to be further enriched to obtain a precise concentration of platelets. Meanwhile, the gelling state of PRFM limits its use in injection therapy. In contrast, t-PRP can be adjusted freely to obtain the required concentration of platelets and can be maintained in a liquid state for at least 24 h at 4°C before activation. However, the maximum platelet concentration and the number and stability of platelets under long term hypothermic storage for t-PRP still require further experimental verification.

**Figure 8:**
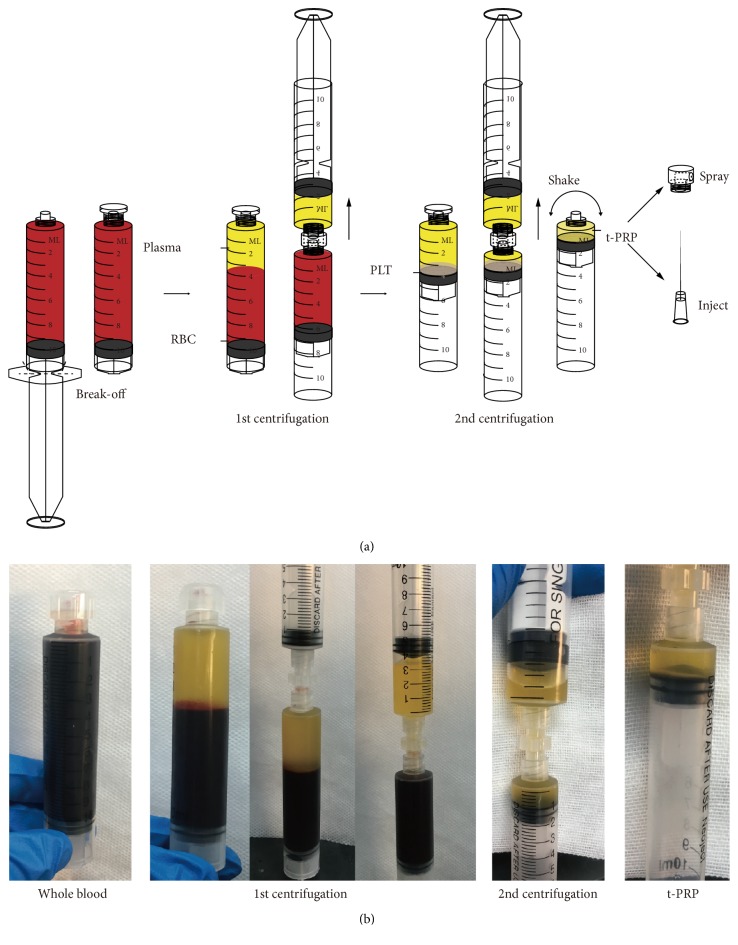
*A clinical usability method for preparing t-PRP*. (a) Illustration for understanding the procedure of the clinical usability t-PRP preparation method. (b) The appearance of the entire t-PRP preparation process.

In summary, t-PRP is a novel and convenient method for preparation and activation of PRP without any additives. Compared to c-PRP, t-PRP reflects more natural and physiologic characteristics while ensuring a high quality. At the same time, in addition to the classical laboratory preparation methods, we also provided a clinical-usable t-PRP preparation method which is more affordable with better results compared to other commercial kits (Figure 8). This method may further improve the safety and outcomes in clinical use.

## Figures and Tables

**Table 1:** Platelet (PLT) counts and pH detection in PRP enrichment procedure.

	Whole blood		Plasma		PRP	
	t-PRP	c-PRP	t-PRP	c-PRP	t-PRP	c-PRP
Amount of PLT	1758 ± 210	1446 ± 179	1466 ± 125	1077 ± 111	1156 ± 114	713.8 ± 83.2
Concentration of PLT	175.8 ± 21.0	144.6 ± 17.9	488.7 ± 41.6	307.7 ± 31.8	1156 ± 114	713.8 ± 83.2
Relative concentration^1^	1	0.82 ± 0.04	2.78 ± 0.21	1.75 ± 0.47	6.58 ± 0.45	4.06 ± 0.55
pH	7.48 ± 0.64	7.10 ± 0.83	7.46 ± 0.74	7.08 ± 0.78	7.46 ± 0.52	7.07 ± 1.22

^1^Relative concentration of PLT to untreated whole blood.
